# Passive E06 IgG reduces lesional PC-OxPL and macrophage accumulation in cholesterol-fed Ldlr^−/−^ mice

**DOI:** 10.1016/j.athplu.2026.100615

**Published:** 2026-09-24

**Authors:** Brian Trausch-Quiróz, Alexandre Normand, Xuchu Que, Elena Alekseeva, Xiaohong Yang, Craig Resch, Amir Ravandi, Joseph L. Witztum, Sotirios Tsimikas

**Affiliations:** a Vascular Medicine Program, Division of Cardiovascular Medicine, University of California San Diego, La Jolla, CA, USA; b Division of Endocrinology and Metabolism, University of California San Diego, La Jolla, CA, USA; c Department of Physiology and Pathophysiology, Rady Faculty of Health Sciences, University of Manitoba, Canada; d Institute of Cardiovascular Sciences, St. Boniface Hospital Albrechtsen Research Centre, Winnipeg, MB, Canada; e Section of Cardiology, Department of Internal Medicine, Rady Faculty of Health Sciences, University of Manitoba, Canada; f Precision Cardiovascular Medicine Group, St. Boniface Hospital Albrechtsen Research Centre, Winnipeg, MB, Canada

**Keywords:** Oxidized phospholipids, Oxidation-specific epitopes, E06 IgG, Passive immunization, lipoprotein(a), Atherosclerosis

## Abstract

**Background:**

Oxidized phospholipids (OxPL) promote vascular inflammation and atherosclerosis. Although transgenic expression of the E06 single-chain antibody reduces atherosclerosis in mice, the therapeutic potential and mechanism of passive immunization with full-length recombinant E06 IgG remain unknown.

**Methods:**

Male *Ldl*^*r*−/−^ mice fed a cholesterol-enriched diet received placebo or recombinant E06 IgG (5 or 25 mg/kg subcutaneously twice weekly) for 12 weeks. Plasma lipids, oxidation-specific biomarkers, atherosclerosis, lesional phosphocholine-containing OxPL (PC-OxPL), macrophage content, collagen, and necrotic core were quantified. To assess whether E06 IgG depletes circulating OxPL, apo(a)- and lipoprotein(a)-transgenic mice received a single intravenous injection of E06 IgG followed by serial measurements of oxidation-specific biomarkers and total plasma OxPL by targeted LC–MS/MS.

**Results:**

Terminal plasma E06 IgG concentrations confirmed dose-dependent systemic exposure. Compared with placebo, E06 IgG significantly reduced lesional macrophage content (47.6 ± 14.5% vs 34.4 ± 9.2% vs 34.3 ± 9.1% of lesion area; *P* = 0.013) and lesional PC-OxPL (28.5 ± 8.8% vs 18.2 ± 7.5% vs 20.1 ± 7.0%; *P* = 0.010) in placebo, 5 mg/kg, and 25 mg/kg groups, respectively. No significant differences were observed in plasma cholesterol, triglycerides, circulating OxPL-related biomarkers, aortic root lesion area, plaque collagen, or necrotic core. Acute E06 IgG administration demonstrated target engagement in apo(a)- and Lp(a)-transgenic mice but produced only transient, non-significant changes in circulating OxPL measured by ELISA and LC–MS/MS.

**Conclusions:**

These findings provide proof-of-concept that passive E06 IgG selectively neutralizes lesional PC-OxPL and reduces plaque macrophage accumulation despite minimal sustained effects on circulating OxPL, supporting further investigation of OxPL-targeted therapies and suggesting that circulating OxPL may not adequately reflect vascular target engagement.

## Highlights

• Passive E06 antibody treatment reduced lesional oxidized phospholipids.
• E06 treatment reduced macrophage accumulation in atherosclerotic plaques.
• Lesional effects occurred without sustained depletion of circulating oxidized lipids.
• Both E06 doses produced similar vascular effects despite higher exposure at 25 mg/kg.
• Circulating oxidized phospholipids may not reflect vascular target engagement.

## Graphical abstract

**Figure:**
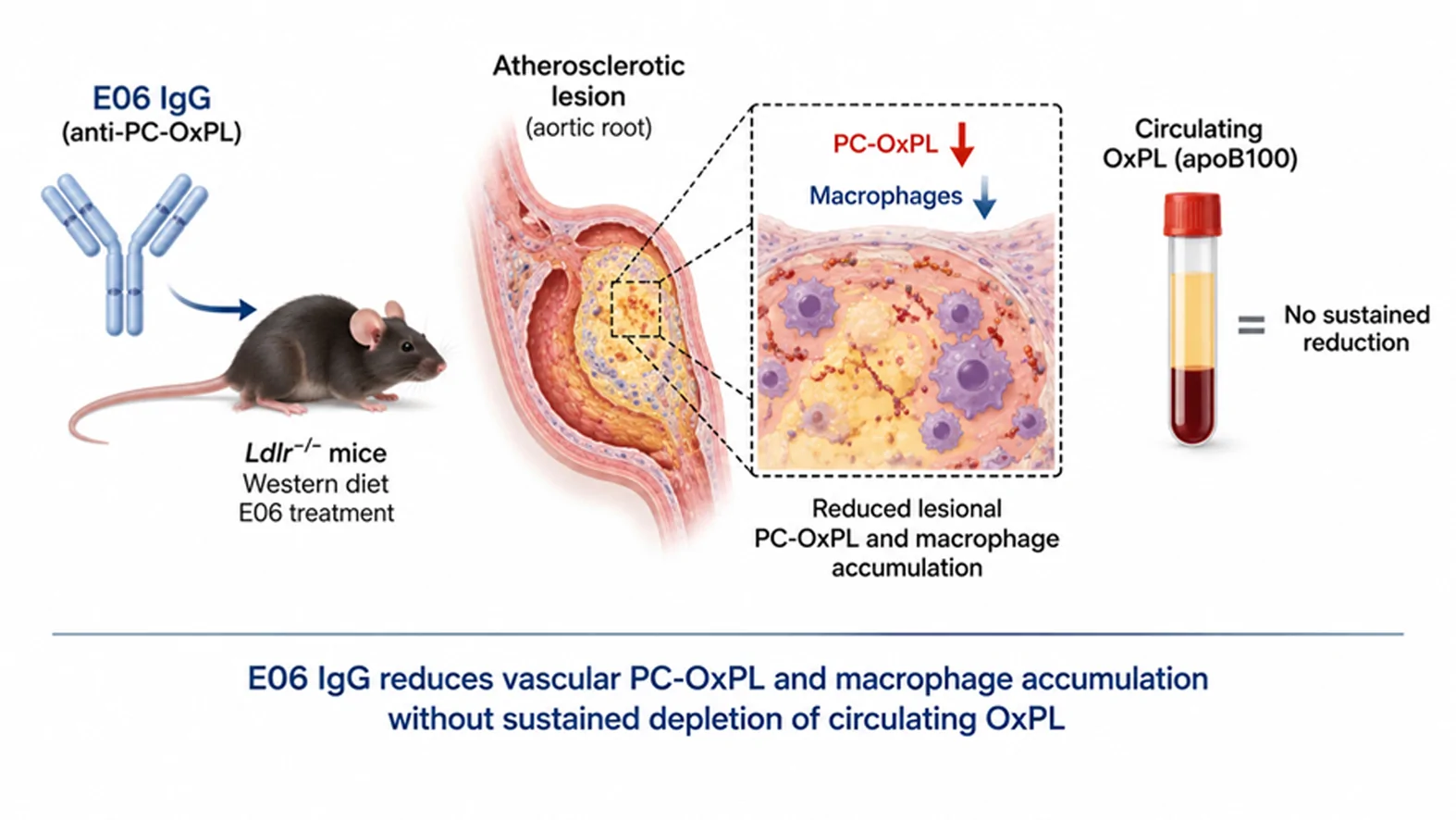

## 1 Introduction

Oxidized phospholipids (OxPL) are generated during oxidative modification of phospholipid-containing lipoproteins and cell membranes and are increasingly recognized as key mediators of vascular inflammation [1]. OxPL accumulate within atherosclerotic plaques, promote endothelial activation, monocyte recruitment, macrophage foam cell formation, and necrotic core expansion, and contribute to multiple stages of atherogenesis. In humans, OxPL are preferentially carried on lipoprotein(a) [Lp(a)] [2,3], where they represent an important determinant of the proatherogenic and proinflammatory properties of Lp(a). Consequently, OxPL have emerged as both biomarkers and potential therapeutic targets for atherosclerotic cardiovascular disease [[4], [5], [6]].

Among the best-characterized oxidation-specific epitopes are phosphocholine-containing OxPL (PC-OxPL), which are recognized by the natural IgM murine monoclonal antibody E06 [6]. E06 binds the PC headgroup of oxidized, but not native, phospholipids and blocks multiple pro-inflammatory effects of PC-OxPL, including macrophage uptake of oxidized LDL, endothelial activation, and inflammatory signaling [[7], [8], [9], [10], [11]]. Genetic expression of E06 single-chain variable fragments (E06-scFv) in hypercholesterolemic mice reduces atherosclerosis and vascular inflammation, establishing proof-of-concept that targeting PC-OxPL is anti-atherogenic [12,13].

Despite these observations, several important translational questions remain unanswered. First, it is unknown whether passive administration of a full-length recombinant E06 IgG antibody can reproduce the anti-inflammatory effects observed with lifelong transgenic expression of E06-scFv. Second, it is unclear whether E06 acts primarily by depleting circulating PC-OxPL or by neutralizing biologically active oxidation-specific epitopes within the vessel wall. Distinguishing between these mechanisms is important for the future development of therapeutic antibodies targeting OxPL.

We therefore generated recombinant murine E06 IgG and evaluated its therapeutic effects in cholesterol-fed *Ldlr*^*−/−*^ mice. The primary objective of this study was to determine whether passive administration of recombinant E06 IgG achieves vascular target engagement in vivo and whether this is associated with changes in lesional and circulating OxPL.

## 2 Materials and methods

### 2.1 Preparation of recombinant E06 IgG

Recombinant E06 IgG1 was produced in suspension HEK293E-253 cells using endotoxin-free plasmid DNA. All glassware and chromatography systems were rendered endotoxin-free by incubation with 0.1 M NaOH for ≥16 h. Heavy- and light-chain expression plasmids were co-transfected using rPEx™ technology at a defined LC:HC:carrier DNA ratio of 0.09375:0.03125:0.875. Cultures were expanded to 30 L and harvested 6 days later. Conditioned medium was clarified using the SartoClear Dynamics system, concentrated to ∼500 mL, and diafiltrated against 4 L PBS using five 30-kDa Hydrosart ultrafiltration cassettes. Precipitates were removed by centrifugation (4000 × g, 10 min) followed by 0.8-μm and 0.45-μm filtration.

Antibody purification was performed using two sequential runs on a 35-mL MabSelectSure LX Protein A column. Bound material was washed with PBS, PBS + 1 M NaCl, and PBS, and eluted with 20 mM citrate, 150 mM NaCl (pH 3.0), followed by immediate neutralization with 1 M K_2_HPO_4_/KH_2_PO_4_ (pH 8.0). Pooled fractions were concentrated to 45 ± 5 mL using 30-kDa centrifugal filters, sterile-filtered (0.22 μm), and further purified by Superdex 200 50/1000 size-exclusion chromatography. Final preparations were formulated in PBS at 20 ± 0.5 mg/mL, aliquoted, snap-frozen in liquid nitrogen, and stored at −80°C. Purity and molecular integrity were confirmed using LabChip Protein Clear HR capillary electrophoresis under reducing and non-reducing conditions. Endotoxin levels were 0.02 EU/mL, and final purity exceeded 98%. Two independent lots (5453 and 5205) exhibited similar potency, with EC_50_ values of 0.360 nM and 0.404 nM, respectively (Supplementary Fig. 1). The antibody was formulated in sterile PBS for in vivo subcutaneous injection studies in *Ldlr*^*−/−*^ mice.

### 2.2 Murine models

Male Ldlr−/− mice were initially placed on a high-fat, high-cholesterol induction diet (21% milk fat, 1% cholesterol) for 2 weeks to rapidly induce hypercholesterolemia and initiate atherosclerosis. They were then transitioned to a 0.5% cholesterol-enriched diet for the 12-week treatment period to maintain an ongoing atherogenic stimulus while reducing prolonged exposure to the more severe high-fat, high-cholesterol diet. This dietary strategy was modeled after our prior passive immunization study with the oxidation-specific antibody IK17-Fab in Ldlr−/− mice [14], thereby facilitating comparison with previous therapeutic studies targeting oxidation-specific epitopes. Thus, the intervention was initiated during early lesion development rather than after establishment of advanced atherosclerotic lesions.

The mice were randomized 1:1:1 to receive E06 IgG at 5 mg/kg, 25 mg/kg, or placebo (75 μL sterile 1× PBS, prepared by dilution of 10× PBS) administered subcutaneously twice weekly for 12 weeks. Mice were weighed weekly and dosed on consistent schedules. Plasma was collected at five timepoints: pre-induction, baseline prior to the 0.5% cholesterol diet, and at 1, 2, and 3 months of treatment. At study termination, mice were euthanized by CO_2_ inhalation, and atherosclerosis was quantified in the aorta *en face* and aortic root.

For acute injection studies, both male and female human apo(a) and Lp(a) transgenic mice on a standard chow diet as previously described [[15], [16], [17]] were used. These models were selected because they express high circulating concentrations of human apo(a) and Lp(a), which are associated with markedly elevated plasma PC-OxPL. In contrast, *Ldlr*^−/−^ mice contain relatively low circulating PC-OxPL despite developing advanced hypercholesterolemia and atherosclerosis. The apo(a) and Lp(a) transgenic models therefore provided an experimental system to directly test whether acute administration of E06 IgG reduces circulating PC-OxPL in vivo under conditions of high PC-OxPL burden. Apo(a) and Lp(a) transgenic mice received a single intravenous injection of 1 mg E06 IgG via the tail vein. Blood samples were collected immediately before antibody administration (baseline) and at 15 min, 3 h, 6 h, and 24 h after injection. Plasma was isolated by centrifugation and stored at −80°C until subsequent lipidomic analyses.

### 2.3 Atherosclerosis quantification

Slides were imaged using an Olympus VS200 slide scanner (20× objective) at the University of California San Diego Microscopy Core Facility. All samples were acquired using identical exposure settings and scanning parameters. Quantitative image analysis was performed using ImagePro 6.0, which was used to delineate aortic root lesion areas and marker-positive area was expressed as a percentage of total lesion area for biotinylated E06 (PC-OxPL) and CD68^+^ macrophages.

### 2.4 Aorta en face lesion area

Aortas were harvested at study termination, split, pinned and Sudan-stained for presence of atherosclerotic plaques, as described previously [12]. Atherosclerosis was then quantified with automated computer-assisted image analysis by measuring lesion area and dividing by total aorta *en face* area, generating an atherosclerosis percentage. This quantification was performed by 3 investigators blinded to treatment assignment with pooling and averaging of the data.

### 2.5 Aortic root lesion area

Aortic root cross-sectional atherosclerosis was measured by cutting successive 10 μm paraffin sections from the origin of the aortic valve, determined as the first section to show a portion of valve leaflet, until the last leaflet shown in a section. This process generated from 70 to 80 sections in each mouse. Modified van Gieson elastic stain was used to enhance differentiation of the intima from surrounding tissue. Quantitative analysis of lesion area was performed on every tenth section, with a total of 7 sections analyzed, spanning 700 μm from the origin of the first visible leaflet. The results are presented as total lesion area in mm^2^ of all aortic cross sections analyzed. Quantification was performed by investigators blinded to treatment assignment using computer-assisted image analysis.

### 2.6 Immunohistochemistry

Alternate aortic root sections were stained for macrophages, PC-OxPL and collagen. Macrophages were stained using rabbit anti-CD68 (Abcam ab125212; 1 μg/mL) with Alexa Fluor 647 goat anti-rabbit secondary antibody (A31634; 5 μg/mL). PC-OxPL epitopes were detected using biotinylated E06 (100 μg/mL) followed by alkaline phosphatase (VECTASTAIN AK-5000) and Vector Red fluorescence using the appropriate fluorescence channel. Collagen staining followed standard protocols using Masson's Trichrome for visualization. For all stains, ImagePro 6.0 was used to quantify total lesion area and the percentage of lesion area positive for each marker.

### 2.7 Immunofluorescence of macrophage and PC-OxPL staining of aortic root sections

Immunofluorescence staining for macrophages and PC-OxPL was performed on paraffin-embedded aortic root sections. For CD68 macrophage staining, sections were deparaffinized and subjected to antigen retrieval in sodium citrate buffer at 95°C for 30 min. After cooling, sections were blocked for 30 min at room temperature and incubated overnight at 4°C with a rabbit polyclonal anti-CD68 antibody (Abcam, ab125212; 1 μg/mL). Following PBS washes, slides were incubated for 1 h with goat anti-rabbit Alexa Fluor 647 secondary antibody (A31634; 5 μg/mL), washed again in PBS, rehydrated, mounted, and imaged.

For PC-OxPL staining, deparaffinization and antigen retrieval were performed as above. Sections were blocked for 30 min and incubated overnight with biotinylated IgM E06 antibody (100 μg/mL). After TBS-T washes, slides were incubated with alkaline phosphatase reagent (VECTASTAIN® AK-5000), washed, and developed for 30 min using Vector® Red substrate (SK-5100). Sections were then rinsed in TBS-T, counterstained with hematoxylin for 30 s, washed in tap water until clear, rehydrated, mounted, and imaged.

### 2.8 Terminal plasma E06 levels

Plasma E06 IgG concentrations were measured at Charles River Laboratories using a PC-BSA ELISA. Plates coated with PC-BSA (5 μg/mL) captured circulating E06, which was detected using an anti-murine Fc antibody. Concentrations were determined using a standard curve of recombinant E06 IgG and reported as nmol/L.

### 2.9 Total cholesterol and triglyceride measurements

Total cholesterol and triglycerides were measured at −2 weeks, baseline, and weeks 4, 8, and 12. Plasma was diluted 1:50 and assayed using enzymatic reagents (Pointe Scientific). Absorbance was measured at 500 nm with background subtraction at 700 nm using a BioTek Gen5 microplate reader.

### 2.10 Oxidation-specific epitopes chemiluminescent immunoassays

Chemiluminescent ELISAs were performed for six oxidation-related biomarkers, OxPL-apoB, MDA-mouse apoB, IgG/IgM to the MDA mimotope, and IgG/IgM to PC-BSA, using previously validated methods. For PC-OxPL detection assays, biotinylated E03 (b-E03) was used. E03 [7] is sequence-identical to E06 and has the same PC-OxPL binding specificity but is preferentially used as the biotinylated detection reagent because of its more efficient and reproducible biotinylation.

**OxPL-apoB:** OxPL-apoB on mouse apoB-100 was measured by capturing murine apoB-100-containing lipoproteins (1:50 plasma dilution) on microtiter plates coated with monoclonal antibody LF5 (5 μg/ml) directed to mouse apoB-100 [18], followed by detection with 1 μg/ml IgM biotinylated E03. A standard curve of phosphocholine equivalents was used to derive OxPL-apoB in nmol/L [1,19].

**MDA-mouse apoB:** Malondialdehyde (MDA) epitopes on mouse apoB were measured in a similar manner as OxPL-apoB, except the plasma dilution was 1:10. Bound LA25 (2.5 μg/ml) was detected with a murine anti-hemagglutinin alkaline phosphatase-conjugated monoclonal antibody [20]. The detecting antibody was a murine anti-hemagglutinin alkaline phosphatase-conjugated monoclonal antibody (Sigma-Aldrich).

**IgG and IgM autoantibodies to phosphocholine-modified bovine serum albumin (PC-BSA):** Antibodies binding PC-BSA were measured in an analogous fashion by plating (5 μg/ml) and detecting with goat anti-mouse IgG and IgM secondary antibodies.

**IgG and IgM autoantibodies to malondialdehyde (MDA) peptide mimotope**: An 11 amino acid linear peptide mimotope to MDA was previously described that immunologically mimics MDA-derived oxidation-specific epitopes [21]. In brief, the MDA mimotope (5 μg/ml) was coated on microtiter well plates, mouse plasma added at 1:10 dilution and the presence of IgG or IgM antibodies binding the MDA mimotope was detected with goat anti-mouse IgG or IgM secondary antibodies (Sigma-Aldrich) as previously described [[22], [23], [24]]**.**

### 2.11 Acute E06 IgG injection studies in apo(a)- and Lp(a)-transgenic mice

To evaluate the acute effects of recombinant E06 IgG on circulating lipoproteins and oxidation-specific epitopes, separate cohorts of apo(a)-transgenic (n = 3) and Lp(a)-transgenic (n = 4) mice were administered a single intravenous dose of E06 IgG (1 mg per mouse). Blood samples were obtained immediately before antibody administration (baseline) and at 15 min, 3 h, 6 h, and 24 h after injection.

Plasma concentrations of Lp(a), apo(a), apo(a)-mouse apoB complexes, OxPL-apo(a), OxPL-apoB, and OxPL-mouse apoB were measured using previously described chemiluminescent ELISAs employing monoclonal antibodies specific for human apo(a), human apoB-100, or mouse apoB, together with biotinylated E06 for detection of PC-OxPL [3,16,17]. Circulating recombinant E06 IgG was quantified with a modified capture ELISA, as previously described for E06-scFv [12], by plating PC-BSA at 5 μg/ml, adding plasma and detecting with alkaline phosphate-conjugated rabbit anti-mouse IgG, using a standard curve of purified E06 IgG as the calibrator. To determine the presence of injected E06 binding to apo(a)/Lp(a), the anti-idiotype monoclonal antibody AB1-2 [25], which specifically recognizes E06 bound to its target, was used. In brief, monoclonal antibody LPA4 was used to capture apo(a)/Lp(a), and biotinylated AB1-2 was used to detect particle-associated E06. Because AB1-2 recognizes the E06 antigen-binding region, detection requires at least one accessible E06 combining site and may therefore underestimate total particle-bound E06. Therefore, the values likely underestimate the true concentration of E06 bound to apo(a)/Lp(a).

### 2.12 Quantification of oxidized phospholipids by LC-MS/MS

Oxidized phospholipids (OxPL) were quantified in plasma using our previously validated DNPH derivatization liquid chromatography-tandem mass spectrometry (LC-MS/MS) assay, which simultaneously measures 60 individual OxPL species across five phospholipid classes, including phosphatidylcholine (PC), phosphatidylethanolamine (PE), phosphatidylinositol (PI), phosphatidylglycerol (PG), and phosphatidylserine (PS), as previously described [26]. Peak areas were normalized to the corresponding class-specific internal standard; because a PI internal standard was not included, OxPI species were normalized to the PE internal standard.

*DNPH derivatization-* Plasma (50 μL) was mixed with an equal volume of 2,4-dinitrophenylhydrazine (DNPH; 5 mg/mL in 1 N HCl), protected from light, and incubated at 37°C for 1 h before immediate lipid extraction.

Lipid extraction- Lipids were extracted using the Folch method. Briefly, 100 μL of the derivatized sample was added to 1 mL chloroform:methanol (2:1, v/v), 90 μL phosphate-buffered saline, and phospholipid class-specific internal standards (0.3 nmol/mL each): PC 13:0/13:0, PE 17:0/17:0, PS 17:0/17:0, and PG 17:0/17:0. After phase separation by centrifugation, the organic phase was collected, evaporated under nitrogen, reconstituted in butanol:methanol (1:1, v/v), and stored at −80°C until analysis.

*Liquid chromatography***-** Chromatographic separation was performed on a Zorbax RRHD Eclipse Plus C18 column (2.1 × 50 mm, 1.8 μm; Agilent) using a Shimadzu Prominence UFLC system at 50°C and a flow rate of 300 μL/min. Mobile phase A consisted of tetrahydrofuran:methanol:water (20:20:60, v/v/v) and mobile phase B of tetrahydrofuran:methanol:water (75:20:5, v/v/v), each containing 10 mmol/L ammonium formate. Following injection of 10 μL sample, solvent B was increased linearly from 0% to 100% over 8 min, maintained for 2.5 min, and returned to initial conditions over 30 s, for a total run time of 11.1 min.

*Mass spectrometry***-** The HPLC system was coupled to an AB Sciex 4000 QTRAP triple quadrupole mass spectrometer equipped with a Turbo V electrospray ionization source operating in positive ion mode. OxPL-DNPH species were quantified using scheduled multiple reaction monitoring (sMRM) with 60 analyte-specific transitions. Samples were analyzed in triplicate.

For all analytes, the precursor ion (Q1) corresponded to the parent OxPL mass plus the 180-Da DNPH adduct. Product ion transitions were optimized for each phospholipid class: OxPC (*m*/*z* 184.3), OxPE (neutral loss of 141 Da), OxPI (neutral loss of 277 Da), OxPS (neutral loss of 185 Da), and OxPG (neutral loss of 189 Da). Compound-specific instrument parameters are provided in Supplementary Table 1. Scheduled retention time windows of ±15 s were used for all transitions.

Data acquisition was performed using Analyst version 1.6 (AB Sciex), and quantification was carried out with MultiQuant version 2.1 (AB Sciex). Peak areas were normalized to class-specific internal standards, with the PE internal standard used for quantification of OxPL species.

### 2.13 Statistical analysis

Continuous variables are presented as mean ± SD unless otherwise indicated. For the E06 IgG treatment study in *Ldlr*^*−/−*^ mice, comparisons among placebo, 5 mg/kg E06 IgG, and 25 mg/kg E06 IgG groups were performed using one-way analysis of variance (ANOVA). When the overall ANOVA was significant, pairwise comparisons were performed using Tukey's multiple-comparison test. Longitudinal plasma biomarker measurements were analyzed at each time point by one-way ANOVA, and treatment effects over time were additionally evaluated using repeated-measures analysis where appropriate. Within each transgenic model, post-injection values were compared with baseline using paired two-tailed t tests. Correlations between circulating E06 IgG concentrations and tissue endpoints were assessed using Spearman rank correlation coefficients. Associations between lesional PC-OxPL and macrophage area were assessed using Pearson correlation coefficients. Apo(a)- and Lp(a)-transgenic mice were analyzed separately for immunoassay endpoints because they express biologically distinct circulating particles. Because LC-MS/MS measures total plasma OxPL independently of the carrier particle, the apo(a)-transgenic and Lp(a)-transgenic mice were pooled for the LC-MS/MS analysis (total n = 7). All statistical analyses were performed using SPSS Statistics version 30 (IBM Corp., Armonk, NY). All statistical tests were two-sided, and a *P* value < 0.05 was considered statistically significant.

## 3 Results

### 3.1 Effect of E06 IgG on total cholesterol and triglycerides

Total cholesterol and triglyceride levels rose sharply during dietary induction and then declined after the switch to the 0.5% cholesterol diet, stabilizing thereafter. The cholesterol and triglyceride levels were not significantly different between the groups at any time point (Table 1).

**Table 1:** Total cholesterol and triglyceride levels at the various timepoints in the study.

Total Cholesterol, mg/dL	−2 weeks	Baseline post HC diet initiation	4 Weeks	8 Weeks	12 Weeks
*Ldlr*^*−/−*^ Placebo	656 ± 28.5	2319 ± 47.3	1687 ± 91.2	1465 ± 92.1	1538 ± 70.3
*Ldlr*^*−/−*^ 5 mg/kg E06 IgG	652 ± 21.4	2370 ± 88.5	1664 ± 60.1	1365 ± 105.0	1703 ± 94.0
*Ldlr*^*−/−*^ 25 mg/kg E06 IgG	663 ± 14.6	2228 ± 90.7	1566 ± 80.9	1511 ± 59.9	1688 ± 66.1
**P-Value**	0.920	0.407	0.652	0.546	0.392

Triglycerides, mg/dL	−2 weeks	Baseline post HC diet initiation	4 Weeks	8 Weeks	12 Weeks
*Ldlr*^*−/−*^ Placebo	302 ± 12.1	616 ± 37.1	153 ± 17.7	311 ± 17.2	771 ± 41.6
*Ldlr*^*−/−*^ 5 mg/kg E06 IgG	337 ± 14.1	612 ± 31.9	155 ± 13.2	343 ± 20.3	819 ± 31.2
*Ldlr*^*−/−*^ 25 mg/kg E06 IgG	300 ± 11.1	571 ± 31.6	158 ± 14.0	308 ± 19.9	798 ± 37.7
P-Value	0.098	0.699	0.861	0.440	0.728

Indicated parameters were measured in high cholesterol (HC)-fed *Ldlr*^*−/−*^ mice at pre-bleed (2 weeks before treatment), baseline (day 0), and after 4, 8, and 12 weeks of treatment. Data are presented as mean ± SEM. P values represent overall between-group comparisons at each individual time point by one-way ANOVA. Repeated-measures mixed-effects analysis similarly demonstrated no significant treatment effect or treatment-by-time interaction for either total cholesterol or triglycerides. n = 8 for the *Ldlr*^*−/−*^ placebo group and n = 10 for both the *Ldlr*^*−/−*^ + 5 mg/kg E06 IgG and *Ldlr*^*−/−*^ + 25 mg/kg E06 IgG groups. HC- high fat/high cholesterol.

### 3.2 Effect of E06 IgG on oxidation-specific epitope biomarker levels

Serial plasma measurements demonstrated no significant differences among groups in OxPL-apoB, MDA-apoB, MDA-mimotope IgM, or IgM PC-BSA over the course of the study, indicating that most circulating OxPL-related biomarkers were not materially altered by E06 treatment (Supplementary Table 2). In contrast, MDA-mimotope IgG differed significantly among groups at 4 weeks and 8 weeks, with lower levels at 4 weeks in the E06-treated groups and a significant between-group difference again at 8 weeks.

As expected, plasma titers of IgG PC-BSA increased in a dose-dependent manner, reflecting the presence of circulating E06 IgG (Fig. 1). By 1 month, IgG PC-BSA levels were substantially higher in the 25 mg/kg group, intermediate in the 5 mg/kg group, and unchanged in placebo, and levels remained markedly elevated in both E06-treated groups throughout follow-up.

**Fig. 1:**
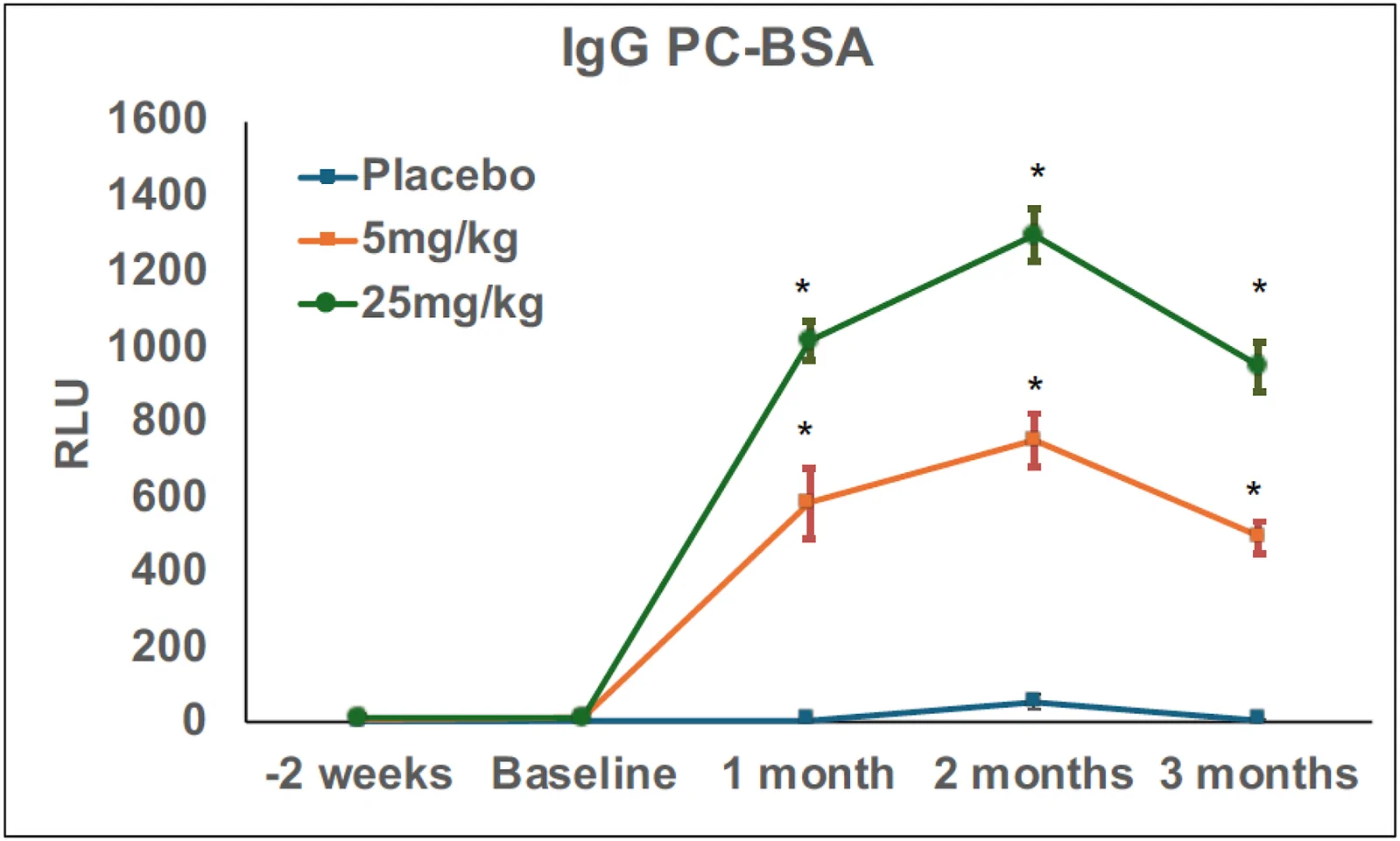
**Plasma IgG antibodies to phosphocholine-modified bovine serum albumin (PC-BSA) following passive E06 IgG immunization.** Plasma IgG antibodies binding PC-BSA were measured by chemiluminescent ELISA at baseline and after 4, 8, and 12 weeks of treatment in placebo-, 5 mg/kg-, and 25 mg/kg-treated *Ldlr*^−/−^ mice. PC-BSA IgG levels increased in a dose-dependent manner, reflecting circulating recombinant E06 IgG. Values are mean ± SD. Statistical comparisons were performed by one-way ANOVA at each time point.

### 3.3 Atherosclerosis quantification

Representative *en face* aortas (Fig. 2A) illustrate lesion formation in vehicle-treated *Ldlr*^*−/−*^ mice and in *Ldlr*^*−/−*^ mice administered E06 IgG subcutaneously at 5 mg/kg or 25 mg/kg for 12 weeks. Quantitative analyses of aortic en face lesions (Fig. 2B) showed similar findings in the placebo and 5 mg/kg E06 IgG groups, with an approximately 20% lower mean lesion area in the 25 mg/kg group (5.50 ± 1.21% vs 6.42 ± 2.34% vs 4.38 ± 0.91% for placebo, 5 mg/kg, and 25 mg/kg, respectively; overall ANOVA P = 0.0337). Post hoc Tukey testing showed a significant difference between the 25 mg/kg and 5 mg/kg groups (P = 0.026).

**Fig. 2:**
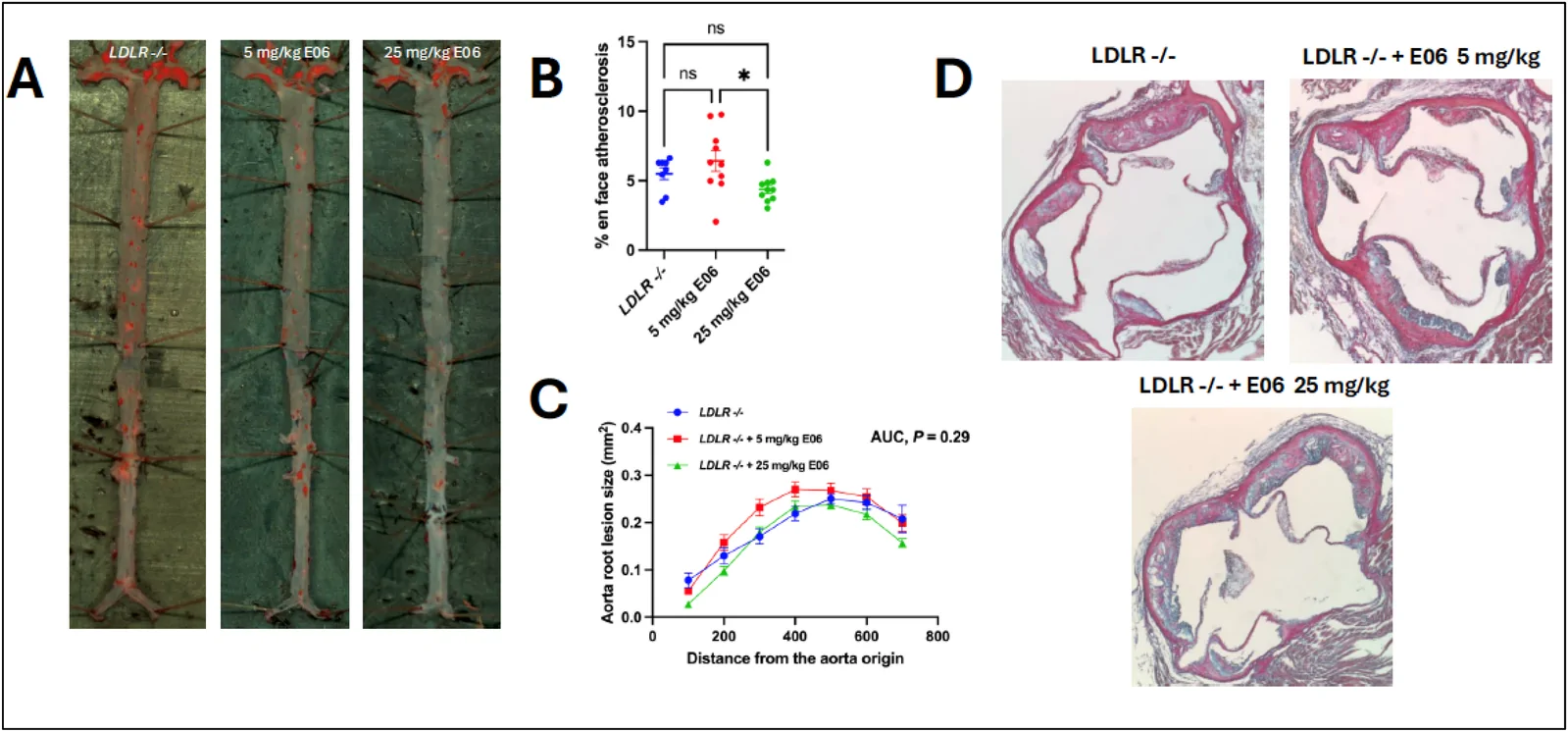
**Effect of passive E06 IgG immunization on aortic atherosclerosis in *Ldlr*^−/−^ mice.** (A) Representative Sudan IV-stained *en face* aortas from placebo-, 5 mg/kg-, and 25 mg/kg-treated mice. (B) Quantification of *en face* lesion area expressed as percentage of total aortic surface. (C) Quantification of % aortic root lesion area. (D) Representative cross-sections of aortic root lesions. Values are mean ± SD. Statistical comparisons were performed using one-way ANOVA with Tukey's post hoc test.

No significant differences were present among groups when atherosclerosis was evaluated as lesion size in mm² versus distance (P = 0.29, Fig. 2C). Representative aortic root lesions are shown in Fig. 2D.

### 3.4 Macrophage content

Quantitative immunostaining demonstrated a significant reduction in macrophage content within aortic root lesions of *Ldlr*^−/−^ mice treated with E06 IgG compared with placebo-treated mice (Fig. 3A). Macrophage-positive area was reduced from 47.6 ± 14.5% of lesion area in placebo-treated mice to 34.4 ± 9.2% in mice treated with E06 IgG at 5 mg/kg and 34.3 ± 9.1% in mice treated with 25 mg/kg (overall ANOVA P = 0.013). Post hoc analysis demonstrated significant reductions in macrophage content for both the 5 mg/kg (P = 0.025) and 25 mg/kg (P = 0.024) treatment groups compared with placebo, whereas there was no difference between the two E06 dose groups (P > 0.99). Representative immunofluorescence images (Fig. 3B) illustrate the marked reduction in lesional macrophage staining following E06 treatment. Furthermore, macrophage content correlated inversely with circulating E06 IgG concentrations in both treatment groups, with significant negative Spearman correlations observed in mice receiving 5 mg/kg (ρ = −0.86, P = 0.0086; Fig. 3C) and 25 mg/kg (ρ = −0.87, P = 0.0029; Fig. 3D).

**Fig. 3:**
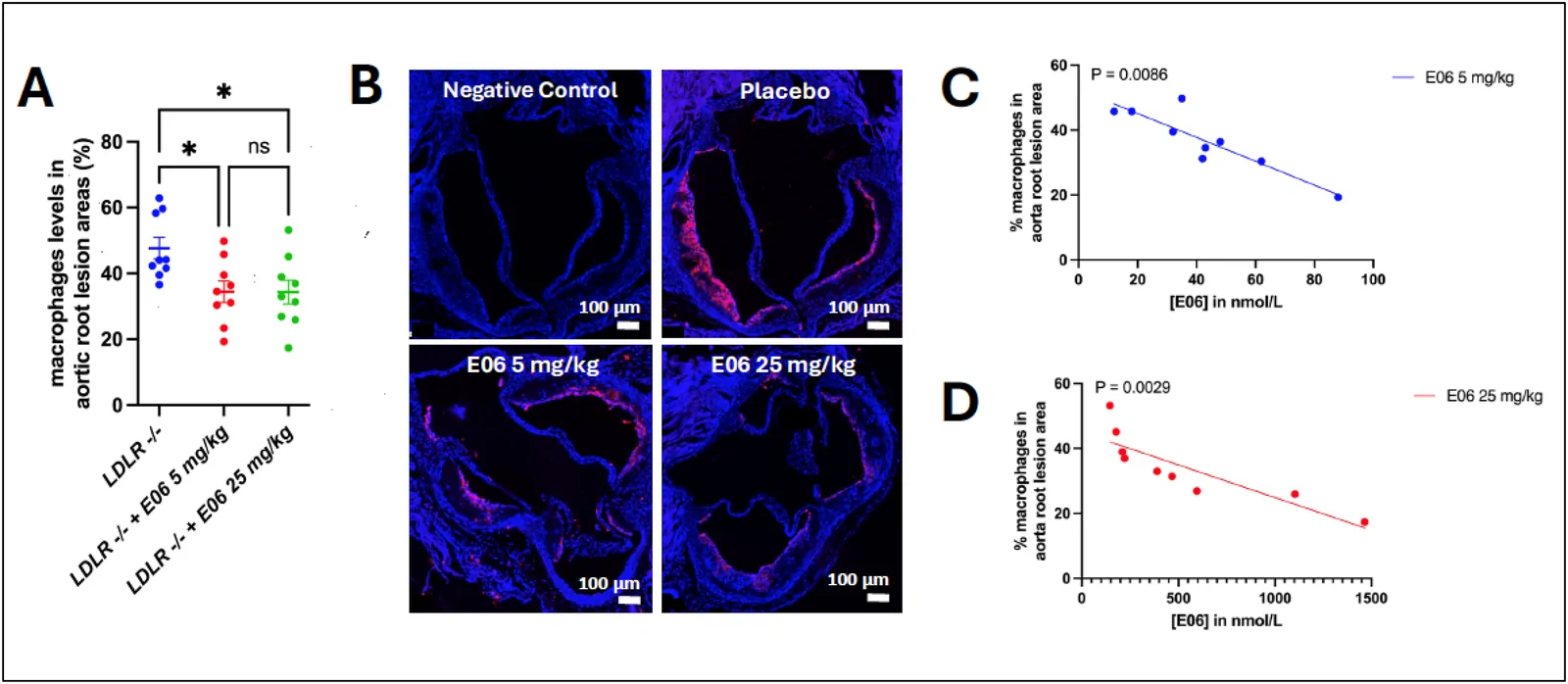
**E06 IgG reduces lesional macrophage content in aortic root plaques.** (A) Quantification of CD68-positive macrophage area expressed as percentage of total plaque area. (B) Representative immunofluorescence images of CD68 staining. (C-D) Correlation between circulating E06 IgG concentrations and lesional macrophage content in mice receiving 5 mg/kg (C) and 25 mg/kg (D) E06 IgG. Values are mean ± SD. Correlations were assessed using Spearman rank analysis.

### 3.5 PC-OxPL area

Quantitative immunohistochemical analysis demonstrated a significant reduction in PC-OxPL accumulation within aortic root lesions of *Ldlr*^*−/−*^ mice treated with E06 IgG compared with placebo-treated mice (Fig. 4A). PC-OxPL-positive area was reduced from 28.5 ± 8.8% of lesion area in placebo-treated mice to 18.2 ± 7.5% in mice treated with E06 IgG at 5 mg/kg and 20.1 ± 7.0% in mice treated with 25 mg/kg (overall ANOVA *P* = 0.010). Post hoc analysis demonstrated significantly lower lesional PC-OxPL content in both the 5 mg/kg (*P* = 0.012) and 25 mg/kg (*P* = 0.038) treatment groups compared with placebo, whereas there was no difference between the two E06 dose groups (*P* = 0.74). Representative immunofluorescence images (Fig. 4B) demonstrate the marked reduction in PC-OxPL staining within atherosclerotic lesions following E06 treatment. Furthermore, lesional PC-OxPL content correlated inversely with circulating E06 IgG concentrations in both treatment groups, with significant negative Spearman correlations observed in mice receiving 5 mg/kg (ρ = −0.93, *P* = 0.0004; Fig. 4C) and 25 mg/kg (ρ = −0.88, *P* = 0.0013; Fig. 4D).

**Fig. 4:**
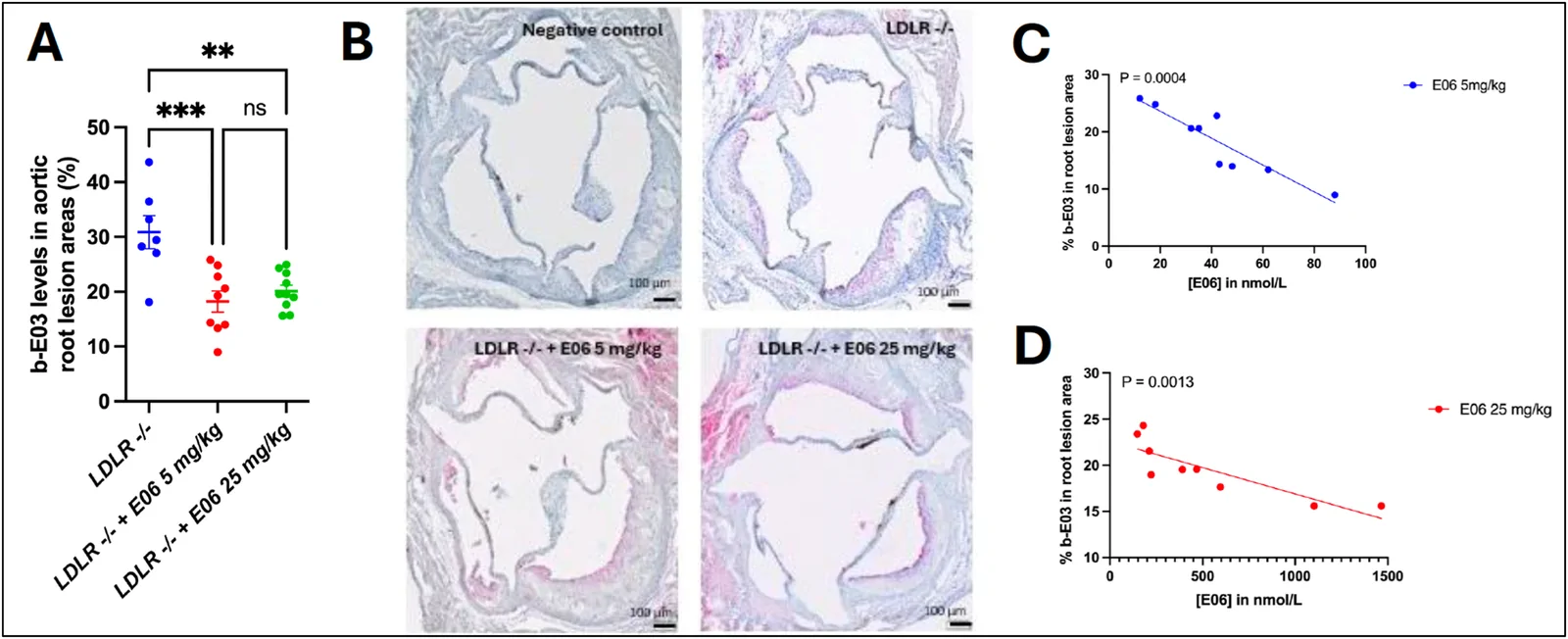
**E06 IgG reduces lesional PC-OxPL epitopes**. (A) Quantification of PC-OxPL-positive area in aortic root lesions detected using biotinylated E03 and expressed as percentage of total plaque area. (B) Representative fluorescence images demonstrating PC-OxPL staining. (C-D) Correlation between circulating E06 IgG concentrations and lesional PC-OxPL content in mice receiving 5 mg/kg (C) and 25 mg/kg (D) E06 IgG. Values are mean ± SD. Correlations were assessed using Spearman rank analysis. b-E03 denotes biotinylated E03, an antibody clone identical to E06 used to detect lesional PC-OxPL.

### 3.6 Collagen

Quantitative analysis of Masson's trichrome staining demonstrated no significant differences in collagen content within aortic root lesions among treatment groups. Collagen-positive area comprised 47.5 ± 13.4% of lesion area in placebo-treated mice, 45.3 ± 7.9% in mice treated with E06 IgG at 5 mg/kg, and 44.7 ± 10.0% in mice treated with 25 mg/kg (overall ANOVA *P* = 0.872).

### 3.7 Necrotic core

The mean necrotic core burden was 4.71% ± 2.32% in placebo mice, 4.72% ± 2.00% in mice treated with E06 at 5 mg/kg, and 5.79% ± 3.09% in mice treated with E06 at 25 mg/kg. Comparison across groups by one-way ANOVA showed no significant difference in necrotic core burden among the three groups (P = 0.631).

### 3.8 Terminal circulating E06 IgG concentrations

Plasma E06 IgG concentrations measured at the end of the study demonstrated clear dose-dependent systemic exposure. Mean circulating E06 concentrations were 41 ± 22 nM in mice treated with 5 mg/kg E06 IgG and 531 ± 461 nM in mice treated with 25 mg/kg E06 IgG, representing an approximately 13-fold higher exposure in the high-dose group. Despite substantial inter-animal variability in circulating antibody concentrations, particularly in the 25 mg/kg group, both dose levels achieved readily detectable systemic exposure. Across all E06-treated mice, circulating E06 concentrations were strongly associated with plasma PC-BSA IgG levels at 12 weeks (Spearman ρ = 0.73, *P* < 0.001), consistent with a robust pharmacodynamic response to antibody administration.

### 3.9 Relationship between lesional PC-OxPL and macrophage accumulation

Across all animals, lesional PC-OxPL area correlated positively with macrophage area (Pearson *r=*0.547, *P=*0.004) (Supplementary Fig. 2). Similar positive correlations were observed within the E06-treated groups (5 mg/kg: *r* = 0.66, *P=*0.05; 25 mg/kg: *r=*0.70, *P=*0.03), whereas no significant correlation was observed in placebo mice (*r=*0.18, *P=*0.67).

### 3.10 Acute E06 IgG injection demonstrates target engagement without sustained depletion of circulating oxidation-specific epitopes

To determine whether E06 IgG binding altered circulating Lp(a)/apo(a)-associated oxidation-specific epitopes, apo(a)-transgenic (n = 3) and Lp(a)-transgenic (n = 4) mice received a single intravenous injection of recombinant E06 IgG and plasma biomarkers were measured over 24 h (Table 2).

**Table 2:** Effect of acute E06 IgG injection on circulating lipoproteins, oxidation-specific epitopes, and target engagement.

apo(a)-tg mice (n = 3)					
Variable	Baseline	15 min	3 h	6 h	24 h
**Lipoprotein measurements**					
Apo(a), nmol/L	360 ± 34	324 ± 24 (*P* = 0.025)	330 ± 55 (*P* = 0.173)	343 ± 29 (*P* = 0.223)	349 ± 19 (*P* = 0.330)
Apo(a)-mouse apoB, RLU	177,632 ± 30,707	163,033 ± 12,493 (*P* = 0.305)	162,726 ± 21,990 (*P* = 0.158)	152,274 ± 10,885 (*P* = 0.158)	189,890 ± 8654 (*P* = 0.494)
**Oxidation-specific epitopes**					
OxPL-apo(a), nmol/L	174 ± 9	150 ± 3 (*P* = 0.079)	148 ± 15 (*P* = 0.025)	158 ± 9 (*P* = 0.147)	150 ± 6 (*P* = 0.087)
OxPL-mouse apoB, nmol/L	31 ± 15	24 ± 7 (*P* = 0.308)	20 ± 6 (*P* = 0.178)	22 ± 6 (*P* = 0.244)	39 ± 4 (*P* = 0.298)
**Target engagement and circulating E06**					
AB1-2–apo(a), RLU	1326 ± 37	2724 ± 675 (*P* = 0.074)	3447 ± 498 (*P* = 0.016)	2550 ± 389 (*P* = 0.038)	1374 ± 301 (*P* = 0.808)

Lp(a)-transgenic mice (n = 4)					
Variable	Baseline	15 min	3 h	6 h	24 h
**Lipoprotein measurements**					
Lp(a), nmol/L	407 ± 72	371 ± 88 (*P* = 0.082)	382 ± 75 (*P* = 0.009)	384 ± 96 (*P* = 0.279)	422 ± 75 (*P* = 0.039)
apo(a)-mouse apoB, RLU	131,061 ± 23,519	102,472 ± 20,756 (*P* = 0.009)	122,693 ± 18,803 (*P* = 0.207)	105,825 ± 22,865 (*P* = 0.019)	121,509 ± 21,769 (*P* = 0.546)
**Oxidation-specific epitopes**					
OxPL-apoB, nmol/L	142 ± 39	165 ± 42 (*P* = 0.002)	123 ± 31 (*P* = 0.031)	160 ± 34 (*P* = 0.025)	134 ± 37 (*P* = 0.109)
OxPL-apo(a), nmol/L	102 ± 22	124 ± 34 (*P* = 0.057)	108 ± 21 (*P* = 0.054)	113 ± 28 (*P* = 0.054)	113 ± 16 (*P* = 0.051)
OxPL-mouse apoB, nmol/L	47 ± 22	50 ± 20 (*P* = 0.207)	46 ± 26 (*P* = 0.790)	48 ± 15 (*P* = 0.909)	27 ± 8 (*P* = 0.056)
**Target engagement and circulating E06**					
AB1-2–Lp(a), RLU	11,213 ± 700	11,617 ± 1434 (*P* = 0.594)	9672 ± 2030 (*P* = 0.164)	10,850 ± 825 (*P* = 0.622)	5274 ± 1706 (*P* = 0.004)
IgG PC-BSA, RLU	1370 ± 1219	148,785 ± 78,524 (*P* = 0.032)	266,845 ± 40,063 (*P* = 0.001)	271,573 ± 24,549 (*P* < 0.001)	265,781 ± 10,893 (*P* < 0.001)

Values are mean ± SD. *P* values are paired comparisons versus baseline. RLU- relative light units per 100 ms.

In apo(a)-transgenic mice, circulating apo(a) levels decreased transiently at 15 min after injection (360 ± 34 vs. 324 ± 24, *P* = 0.025) but subsequently returned toward baseline. Similarly, apo(a)-mouse apoB complexes showed no significant change during the 24-h observation period. In Lp(a)-transgenic mice, plasma Lp(a) concentrations exhibited modest transient changes after injection, whereas apo(a)-mouse apoB complexes decreased at 15 min (*P* = 0.009) and 6 h (*P* = 0.019) before returning toward baseline at 24 h.

Evidence of rapid antibody target engagement was demonstrated by the anti-idiotype monoclonal antibody AB1-2 present on apo(a). In apo(a)-transgenic mice, AB1-2 binding increased approximately 2.6-fold by 3 h (1326 ± 37 vs. 3447 ± 498, *P* = 0.016) and remained elevated at 6 h (*P* = 0.038), confirming binding of infused E06 IgG to circulating apo(a). In Lp(a)-transgenic mice, AB1-2 levels remained relatively stable during the early time points and declined by 24 h (11,213 ± 700 vs. 5274 ± 1,706, *P* = 0.004), consistent with clearance of antibody-bound Lp(a) complexes. Circulating E06 IgG, measured by the PC-BSA immunoassay, was readily detectable following injection. In Lp(a)-transgenic mice, IgG PC-BSA levels increased markedly within 15 min and remained elevated through 24 h (all *P* ≤ 0.032).

Despite clear evidence of antibody exposure and target engagement, circulating oxidation-specific epitopes showed only modest changes following E06 IgG administration. In apo(a)-transgenic mice, OxPL-apo(a) decreased transiently at 3 h (174 ± 9 vs. 148 ± 15, *P* = 0.025), whereas OxPL-mouse apoB did not change significantly. In Lp(a)-transgenic mice, OxPL-apo(a) remained largely unchanged throughout the study, while OxPL-apoB exhibited modest fluctuations at individual time points without sustained reduction, returning to baseline by 24 h.

### 3.11 Effect of E06 IgG injection on circulating total OxPL species by LC-MS/MS

To determine whether E06 IgG altered the total circulating pool of oxidized phospholipid molecular species beyond the immunologically detectable PC-containing epitopes measured by ELISA, plasma samples from the same mice were subsequently analyzed by targeted LC-MS/MS. Following E06 IgG injection, total plasma OxPL demonstrated a consistent but transient decline. Mean total OxPL decreased from 11.4 ± 9.6 nmol/ml at baseline to 5.7 ± 3.5 nmol/ml at 15 min after injection, representing an approximately 50% reduction. Thereafter, total OxPL progressively increased toward baseline values, measuring 7.8 ± 5.9 nmol/ml at 3 h, 9.0 ± 7.4 nmol/ml at 6 h, and 9.9 ± 8.6 nmol/ml at 24 h (Table 3). Despite this reproducible temporal pattern, neither the overall effect of time (repeated-measures ANOVA *P* = 0.636) nor paired comparisons between individual post-injection time points and baseline reached statistical significance (all *P* > 0.18), reflecting the substantial inter-animal variability and limited sample size.

**Table 3:** Total OxPL values measured by LC-MS/MS in *apo(a)-tg* and *Lp(a)-tg* mice injected with 1 mg E06 IgG and followed with serial blood testing over 24 h.

Time after E06 IgG Injection	Total OxPL, nmol/mL, mean ± SD	Paired P vs baseline
Baseline	11.38 ± 9.64	—
15 min	5.72 ± 3.54	0.184
3 h	7.83 ± 5.94	0.405
6 h	8.99 ± 7.37	0.687
24 h	9.86 ± 8.63	0.682

Total plasma OxPL demonstrated an early numerical reduction after E06 IgG injection, with no significant overall effect over time (repeated-measures ANOVA, P = 0.636). Total plasma OxPL measured by LC-MS/MS in pooled apo(a)- and Lp(a)-transgenic mice (n = 7).

Analysis of individual OxPL classes demonstrated similar temporal patterns for OxPC, OxPE, OxPI, and OxPS, with an early numerical decline after E06 IgG administration followed by gradual recovery toward baseline; none of these class-specific changes reached statistical significance (Supplementary Fig. 3). OxPG was not detected in mouse plasma.

## 4 Discussion

This study provides proof-of-concept that passive immunization with recombinant E06 IgG achieves vascular target engagement, characterized by reduced lesional phosphocholine-containing oxidized phospholipids (PC-OxPL) and macrophage accumulation despite minimal effects on plasma lipids, circulating oxidation-specific biomarkers, or plaque composition. Acute E06 IgG administration similarly demonstrated antibody target engagement while producing only transient, non-significant changes in circulating OxPL measured by both immunoassays and LC-MS/MS. Collectively, these findings support a mechanism in which passive E06 IgG primarily neutralizes biologically active oxidation-specific epitopes within the vessel wall rather than producing sustained depletion of circulating OxPL.

The present study extends previous investigations by examining whether passive immunization with recombinant full-length E06 IgG reproduces key biological effects observed with transgenic E06 expression. Because E06 administration began after a short dietary induction period and baseline lesion burden was not directly quantified, these experiments evaluate the effect of passive E06 IgG during atherosclerosis development and progression rather than regression of established advanced lesions. Constitutive expression of an E06 single-chain variable fragment (E06-scFv), which is locally secreted, reduced atherosclerosis and pro-inflammatory responses in hypercholesterolemic mice, providing proof-of-concept that neutralization of PC-OxPL attenuates vascular disease [12]. However, lifelong, local transgenic expression of an antibody fragment differs fundamentally from therapeutic administration of a full-length immunoglobulin. In the present study, passive E06 IgG administration achieved sustained systemic exposure and selectively reduced lesional PC-OxPL and macrophage accumulation without materially altering plasma lipids or most circulating oxidation-specific biomarkers. Importantly, these biological effects occurred despite minimal sustained changes in circulating OxPL, supporting the concept that vascular target engagement can occur independently of measurable depletion of circulating OxPL.

A principal finding of this study is the dissociation between circulating oxidation-specific biomarkers and lesional PC-OxPL biology. Despite significant reductions in lesional PC-OxPL and macrophage accumulation, passive E06 IgG administration produced little or no sustained change in circulating OxPL-apoB, MDA-apoB, MDA-mimotope antibodies, or other oxidation-specific biomarkers. These observations suggest that plasma OxPL measurements may not accurately reflect vascular target engagement or the burden of biologically active oxidation-specific epitopes within the arterial wall. Rather than depleting the circulating pool of OxPL, E06 IgG appears to selectively neutralize accessible PC-OxPL within the plaque microenvironment, thereby attenuating local inflammatory signaling and macrophage recruitment. This interpretation is consistent with the established ability of E06 to inhibit PC-OxPL-mediated endothelial activation, monocyte adhesion, macrophage uptake of oxidized LDL, and downstream inflammatory responses. If confirmed in future studies, these findings suggest that circulating OxPL biomarkers alone may not be sufficient pharmacodynamic markers for therapies directed against oxidation-specific epitopes [6].

To further define the mechanism of E06 IgG in vivo, we performed acute injection studies in apo(a)- and Lp(a)-transgenic mice that express high circulating levels of Lp(a)-associated oxidation-specific epitopes. Anti-idiotype (AB1-2) measurements demonstrated rapid binding of infused E06 IgG to circulating apo(a)/Lp(a), while the PC-BSA assay confirmed circulating antibody exposure. Despite clear target engagement, circulating OxPL-apo(a), OxPL-apoB, and total OxPL molecular species measured by LC-MS/MS showed only modest or transient changes. These findings emphasize the complementary nature of immunologic and lipidomic assays. The E06 immunoassays primarily detect phosphocholine-containing OxPL recognized by the antibody, whereas the LC-MS/MS assay provides a broader comparative assessment of multiple circulating OxPL molecular species independent of antibody recognition. These data suggest that antibody binding and biological neutralization of PC-OxPL do not necessarily require sustained depletion of the total circulating OxPL pool.

Despite the marked difference in systemic exposure between the two E06 IgG doses, the reductions in lesional PC-OxPL and macrophage content were similar at 5 and 25 mg/kg. This apparent plateau may indicate that the lower dose achieved sufficient tissue exposure to substantially neutralize accessible PC-OxPL epitopes, such that higher circulating antibody concentrations provided little additional biological effect. The strong inverse relationships between circulating E06 IgG and both lesional PC-OxPL and macrophage content within each treatment group are consistent with an exposure-related biological effect. However, with only two active doses and substantial inter-animal variability in antibody exposure, the present study cannot define the dose-response relationship or determine whether this plateau reflects target saturation, tissue penetration, or other factors.

Several mechanisms may explain these observations. First, E06 specifically recognizes phosphocholine-containing OxPL rather than the broader spectrum of oxidized phospholipid molecular species measured by LC-MS/MS. Second, antibody binding may functionally neutralize the biological activity of PC-OxPL without promoting rapid removal of the underlying oxidized phospholipid from the circulation, thereby dissociating biological activity from measurable OxPL mass. Third, oxidized phospholipids are continuously generated and exchanged among circulating lipoproteins, extracellular vesicles, cell membranes, and tissues, potentially replenishing the circulating OxPL pool despite ongoing antibody binding. An additional analytical consideration is that dilution and washing during the assay may permit partial dissociation of therapeutic E06 IgG, allowing the higher-avidity pentameric E06 IgM detection antibody to bind the same PC-OxPL epitopes. Finally, because the LC-MS/MS assay provides a comparative assessment of multiple OxPL molecular species rather than a direct measure of biologically active PC-OxPL epitopes, changes in antibody-accessible oxidation-specific epitopes may not necessarily parallel changes in total circulating OxPL abundance.

This study has limitations. First, the relatively short 12-week treatment period may have limited the ability to detect changes in overall plaque burden despite significant reductions in lesional PC-OxPL and macrophage accumulation, and longer-term studies in larger cohorts are needed to determine whether passive E06 IgG therapy promotes plaque regression or stabilization. Second, in the immunostaining studies, PC-OxPL epitopes may have been masked rather than depleted. Nevertheless, the concordant reductions in lesional PC-OxPL and macrophage content together with direct evidence of antibody target engagement support a target-specific biological effect. Third, an isotype-matched IgG control was not included. Accordingly, although the selective reduction in E06-detectable PC-OxPL supports antigen-specific target engagement, we cannot exclude contributions from Fc-mediated immunomodulatory effects that have been reported with other IgG preparations. Fourth, the murine OxPL-apoB assay selectively captures apoB100-containing lipoproteins and therefore does not quantify OxPL carried on apoB48-containing particles. Because apoB48 comprises the N-terminal portion of apoB100 and has no unique protein sequence that permits selective antibody-based capture, apoB48-associated OxPL could not be measured independently with this immunoassay approach. Given the substantial contribution of apoB48-containing lipoproteins in Ldlr−/− mice, the present measurements may therefore underestimate total apoB-associated OxPL and cannot exclude treatment-related changes in OxPL carried by apoB48-containing particles.

## 5 Conclusions

In conclusion, passive immunization with recombinant E06 IgG achieved vascular target engagement characterized by reduced lesional PC-OxPL and macrophage accumulation despite minimal sustained effects on circulating oxidation-specific biomarkers and total plasma OxPL. These findings provide proof-of-concept that local neutralization of oxidation-specific epitopes can modify vascular inflammation independently of measurable depletion of circulating OxPL and suggest that circulating biomarkers alone may not adequately reflect vascular target engagement during OxPL-directed therapies.

## CRediT author statement

Brian Trausch-Quiróz: Conceptualization, Investigation, Methodology, Formal analysis, Visualization, Writing – original draft, Writing – review & editing.

Alexandre Normand: Conceptualization, Investigation, Methodology, Formal analysis, Visualization, Writing – original draft, Writing – review & editing.

Xuchu Que: Investigation, Methodology, Validation, Writing – review & editing.

Elena Alekseeva: Investigation, Methodology, Validation, Writing – review & editing.

Xiaohong Yang: Investigation, Methodology, Validation, Writing – review & editing.

Craig Resch: Investigation, Methodology, Validation, Writing – review & editing.

Amir Ravandi: Investigation, Methodology, Validation, Writing – review & editing.

Joseph L. Witztum: Conceptualization, Supervision, Funding acquisition, Writing – review & editing.

Sotirios Tsimikas: Conceptualization, Funding acquisition, Project administration, Resources, Supervision, Writing – review & editing.

## Ethics declaration

This study was conducted in accordance with the ARRIVE (Animal Research: Reporting of In Vivo Experiments) guidelines. This study was approved by the UC San Diego Institutional Animal Care and Use Committee (IACUC; Approval No. S00058)and were performed in accordance with the National Institutes of Health Guide for the Care and Use of Laboratory Animals.

## Writing process

None

## Sources of support

This work was supported by NHLBI grants R01 HL159156 and HL170224 to ST and 5T32HL086344-15 to BQ-T.

## Declaration of competing interest

The authors declare the following financial interests/personal relationships which may be considered as potential competing interests: ST and JLW are co-inventors and receive royalties from patents owned by University of California San Diego (UCSD) and are co-founders and have an equity interest in Oxitope, LLC and Calluna Pharma. ST and JLW are co-founders of Kleanthi Diagnostics, LLC. The terms of this arrangement have been reviewed and approved by the University of California, San Diego in accordance with its conflict-of-interest policies. ST has a dual appointment at UCSD and Ionis Pharmaceuticals. The other co-authors have nothing to disclose.
