# Supplementary material for: Passive E06 IgG reduces lesional PC-OxPL and macrophage accumulation in cholesterol-fed Ldlr−/− mice
Source: Atheroscler Plus. 2026 Sep 24;66:100615. doi: 10.1016/j.athplu.2026.100615 (PMC13641036; doi:10.1016/j.athplu.2026.100615)
Supplement: Supplementary file 1 [file mmc1.docx]

**E06 IgG reduces lesional PC-OxPL and macrophage accumulation without sustained depletion of circulating OxPL**

Brian Trausch-Quiróz*, Alexandre Normand*, Xuchu Que, Elena Alekseeva,

Xiaohong Yang, Craig Resch, Amir Ravandi, Joseph L Witztum,

Sotirios Tsimikas

* Equal contribution

**Supplementary Table 1:** Mass spectrometry parameters. Q1: Analyte ion m/z, Q3: Fragment ion m/z, RT: Retention time, DP: Declustering potential, CE: Collision energy

| OxPL | Q1 m/z | Q3 m/z | RT | DP | CE |
| --- | --- | --- | --- | --- | --- |
| PC:16:0, C4H5O3 | 760.6 | 184.3 | 5.74 | 125 | 53 |
| PC:16:0, C5H7O3 | 774.6 | 184.3 | 5.84 | 125 | 53 |
| PC:16:0, C6H7O3 | 786.6 | 184.3 | 5.96 | 125 | 53 |
| PC:16:0, C6H9O3 | 788.6 | 184.3 | 5.94 | 125 | 53 |
| PC:18:0, C4H5O3 | 788.6 | 184.3 | 6.14 | 125 | 53 |
| PC:16:0, C5H7O4 | 790.6 | 184.3 | 5.7 | 125 | 53 |
| PC:16:0, C7H9O3 | 800.6 | 184.3 | 6.01 | 125 | 53 |
| PC:16:0, C7H11O3 | 802.6 | 184.3 | 6.04 | 125 | 53 |
| PC:18:0, C5H7O3 | 802.6 | 184.3 | 6.21 | 125 | 53 |
| PC:18:0, C6H7O3 | 814.6 | 184.3 | 6.11 | 125 | 53 |
| PC:16:0, C8H13O3 | 816.6 | 184.3 | 6.14 | 125 | 53 |
| PC:18:0, C6H9O3 | 816.6 | 184.3 | 6.34 | 125 | 53 |
| PC:16:0, C7H9O4 | 816.6 | 184.3 | 5.78 | 125 | 53 |
| PC:16:0, C7H11O4 | 818.6 | 184.3 | 5.9 | 125 | 53 |
| PC:18:0, C7H9O3 | 828.6 | 184.3 | 6.41 | 125 | 53 |
| PC:16:0, C9H15O3 | 830.6 | 184.3 | 6.25 | 125 | 53 |
| PC:16:0, C8H11O4 | 830.6 | 184.3 | 5.82 | 125 | 53 |
| PC:18:0, C8H13O3 | 844.6 | 184.3 | 6.45 | 125 | 53 |
| PC:16:0, C10H17O3 | 844.6 | 184.3 | 5.91 | 125 | 53 |
| PC:18:0, C7H11O4 | 846.6 | 184.3 | 6.26 | 125 | 53 |
| PC:16:0, C11H17O3 | 856.6 | 184.3 | 6.36 | 125 | 53 |
| PC:18:0, C9H15O3 | 858.6 | 184.3 | 6.58 | 125 | 53 |
| PC:18:0, C8H11O4 | 858.6 | 184.3 | 6.26 | 125 | 53 |
| PC:18:0, C10H13O3 | 868.6 | 184.3 | 6.7 | 125 | 53 |
| PC:16:0, C10H17O5 | 876.6 | 184.3 | 6.2 | 125 | 53 |
| PC:16:0, C12H15O4 | 882.6 | 184.3 | 6.66 | 125 | 53 |
| PC:16:0, C12H17O4 | 884.6 | 184.3 | 6.41 | 125 | 53 |
| PC:18:0, C10H13O4 | 884.6 | 184.3 | 6.7 | 125 | 53 |
| PC:16:0, C12H17O5 | 900.6 | 184.3 | 5.17 | 125 | 53 |
| PC:18:0, C12H17O5 | 928.6 | 184.3 | 5.53 | 125 | 53 |
| PE:16:0, C5H7O3 | 732.6 | 591.6 | 5.95 | 110 | 30 |
| PE:16:0, C6H9O3 | 746.6 | 605.6 | 6.22 | 110 | 30 |
| PE:16:0, C7H11O3 | 760.6 | 619.6 | 6.31 | 110 | 30 |
| PE:18:0, C6H9O3 | 774.6 | 633.6 | 6.22 | 110 | 30 |
| PE:16:0, C9H15O3 | 788.6 | 647.6 | 6.35 | 110 | 30 |
| PE:16:0, C10H17O3 | 802.6 | 661.6 | 6.55 | 110 | 30 |
| PE:18:0, C9H15O3 | 816.6 | 675.6 | 6.66 | 110 | 30 |
| PE:18:0, C10H17O3 | 830.6 | 689.6 | 6.71 | 110 | 30 |
| PE:18:0, C11H19O3 | 844.6 | 703.6 | 6.83 | 110 | 30 |
| PS:16:0, C5H7O3 | 776.6 | 591.6 | 5.11 | 105 | 28 |
| PS:16:0, C6H9O3 | 790.6 | 605.6 | 5.37 | 105 | 28 |
| PS:18:0, C5H7O3 | 804.6 | 619.6 | 5.47 | 105 | 28 |
| PS:18:0, C6H9O3 | 818.6 | 633.6 | 5.45 | 105 | 28 |
| PS:16:0, C9H15O3 | 832.6 | 647.6 | 5.64 | 105 | 28 |
| PS:16:0, C10H15O3 | 846.6 | 661.6 | 5.74 | 105 | 28 |
| PS:16:0, C11H17O3 | 858.6 | 673.6 | 5.64 | 105 | 28 |
| PS:18:0, C9H15O3 | 860.6 | 675.6 | 5.85 | 105 | 28 |
| PS:18:0, C10H15O3 | 874.6 | 689.6 | 5.92 | 105 | 28 |
| PS:18:0, C11H15O3 | 886.6 | 701.6 | 5.5 | 105 | 28 |
| PI:16:0, C4H5O3 | 854.6 | 577.6 | 5.03 | 100 | 30 |
| PI:16:0, C5H7O3 | 868.6 | 591.6 | 5.07 | 100 | 30 |
| PI:18:0, C5H7O3 | 896.6 | 619.6 | 5.44 | 100 | 30 |
| PI:16:0, C8H13O3 | 910.6 | 633.6 | 5.53 | 100 | 30 |
| PI:16:0, C9H15O3 | 924.6 | 647.6 | 5.6 | 100 | 30 |
| PI:18:0, C8H13O3 | 938.6 | 661.6 | 5.69 | 100 | 30 |
| PI:18:0, C9H15O3 | 952.6 | 675.6 | 5.8 | 100 | 30 |
| PG:16:0, C5H7O3 | 780.6 | 591.6 | 5.3 | 80 | 30 |
| PG:18:0, C5H7O3 | 808.6 | 619.6 | 5.8 | 80 | 30 |
| PG:16:0, C9H15O3 | 836.6 | 647.6 | 5.66 | 80 | 30 |
| PG:18:0, C9H15O3 | 864.6 | 675.6 | 5.94 | 80 | 30 |
| IS PC 13:0-13:0 [M+H]+ | 650.5 | 184.3 | 6.21 | 100 | 22 |
| IS PE 17:0-17:0 [M+H]+ | 720.6 | 579.5 | 7.48 | 100 | 30 |
| IS PS 17:0-17:0 [M+H]+ | 764.4 | 579.8 | 6.66 | 105 | 28 |
| IS PG 17:0-17:0 [M+NH4]+ | 768.8 | 579.7 | 6.77 | 80 | 30 |

**Supplementary Table 2.** Plasma concentrations of oxidation-specific biomarkers measured by ELISA over the time course of the experiment following treatment with E06 IgG.

| **Timepoint** |  | **Group** |  |  |
| --- | --- | --- | --- | --- |
| -2 weeks | **Placebo** | **5mg/kg** | **25mg/kg** | **P-Values Between Groups** |
| OxPL-ApoB | 2.1 ± 2.1 | 1.2 ± 0.3 | 1.4 ± 0.6 | 0.39 |
| MDA-apoB | 20516 ± 8804 | 17416 ± 4905 | 17053 ± 3662 | 0.46 |
| MDA-mimotope IgG | 20.1 ± 44.4 | 41.9 ± 27.9 | 33.1 ± 61.6 | 0.62 |
| MDA-mimotope IgM | 37.6 ± 106.4 | 130.2 ± 217.7 | 53.5 ± 56.0 | 0.35 |
| PC-BSA-IgG | 0.0 ± 0.0 | 6.9 ± 13.2 | 11.6 ± 18.9 | 0.23 |
| PC-BSA-IgM | 293.6 ± 270.7 | 395.4 ± 381.4 | 496.5 ± 560.0 | 0.62 |
|  |  |  |  |  |
| Baseline | **Placebo** | **5mg/kg** | **25mg/kg** |  |
| OxPL-ApoB | 2.5 ± 0.9 | 2.0 ± 1.0 | 2.0 ± 0.5 | 0.42 |
| MDA-apoB | 36010 ± 6949 | 37015 ± 20693 | 36300 ± 12878 | 0.99 |
| MDA-mimotope IgG | 7.9 ± 20.7 | 11.1 ± 26.3 | 35.8 ± 69.4 | 0.36 |
| MDA-mimotope IgM | 167.4 ± 220.2 | 153.7 ± 215.0 | 195.1 ± 166.4 | 0.90 |
| PC-BSA-IgG | 5.2 ± 14.8 | 11.1 ± 18.4 | 10.7 ± 22.8 | 0.78 |
| PC-BSA-IgM | 286.4 ± 222.6 | 510.8 ± 496.3 | 800.1 ± 451.5 | 0.049 |
|  |  |  |  |  |
| 4 Weeks | **Placebo** | **5mg/kg** | **25mg/kg** |  |
| OxPL-ApoB | 1.8 ± 0.7 | 1.8 ± 0.5 | 1.7 ± 0.4 | 0.93 |
| MDA-apoB | 11878 ± 10827 | 13699 ± 9068 | 9989 ± 8773 | 0.69 |
| MDA-mimotope IgG | 48.8 ± 38.1 | 13.4 ± 19.5 | 1.1 ± 3.5 | <0.001 |
| MDA-mimotope IgM | 373.2 ± 302.5 | 250.6 ± 178.9 | 222.9 ± 209.9 | 0.37 |
| PC-BSA-IgG | 4.0 ± 9.8 | 583.1 ± 265.2 | 1015.7 ± 143.9 | <0.001 |
| PC-BSA-IgM | 1155.2 ± 975.1 | 623.6 ± 481.5 | 818.9 ± 440.7 | 0.24 |
|  |  |  |  |  |
| 8 weeks | **Placebo** | **5mg/kg** | **25mg/kg** |  |
| OxPL-ApoB | 2.1 ± 1.0 | 1.8 ± 0.4 | 1.4 ± 0.5 | 0.11 |
| MDA-apoB | 15906 ± 7638 | 14403 ± 13035 | 12450 ± 7040 | 0.76 |
| MDA-mimotope IgG | 49.9 ± 31.6 | 111.4 ± 90.8 | 34.9 ± 48.0 | 0.032 |
| MDA-mimotope IgM | 338.9 ± 422.2 | 407.3 ± 286.2 | 241.6 ± 164.4 | 0.47 |
| PC-BSA-IgG | 52.5 ± 56.0 | 751.5 ± 200.6 | 1297.8 ± 163.8 | <0.001 |
| PC-BSA-IgM | 984.0 ± 601.2 | 1061.5 ± 565.5 | 857.9 ± 463.7 | 0.70 |
|  |  |  |  |  |
| 12 weeks | **Placebo** | **5mg/kg** | **25mg/kg** |  |
| OxPL-ApoB | 1.9 ± 0.5 | 2.0 ± 0.5 | 2.2 ± 0.6 | 0.39 |
| MDA-apoB | 25643 ± 19893 | 32749 ± 19293 | 20789 ± 13807 | 0.33 |
| MDA-mimotope IgG | 11.8 ± 14.3 | 55.0 ± 53.1 | 41.7 ± 66.5 | 0.22 |
| MDA-mimotope IgM | 549.4 ± 381.1 | 502.8 ± 325.1 | 422.8 ± 181.0 | 0.67 |
| PC-BSA-IgG | 4.1 ± 9.2 | 491.7 ± 120.5 | 946.0 ± 186.2 | <0.001 |
| PC-BSA-IgM | 1780 ± 894 | 1689 ± 2026 | 1273 ± 1018 | 0.72 |

P-Values represent differences between groups analyzed by ANOVA.

**Supplementary Figure 3.** Temporal changes in individual plasma oxidized phospholipid classes following E06 IgG administration. Plasma OxPC (A), OxPE (B), OxPI (C), and OxPS (D) were quantified by targeted LC-MS/MS at baseline and 15 minutes, 3 hours, 6 hours, and 24 hours after a single intravenous injection of E06 IgG in apo(a)- and Lp(a)-transgenic mice. Individual mice are shown with connected observations; summary values are presented as mean ± SEM. Each OxPL class demonstrated an early numerical decline followed by recovery toward baseline, but no class-specific change reached statistical significance. OxPG was not detected in mouse plasma.

| **OxPL class, nmol/mL** | **Baseline** | **15 min** | **3 h** | **6 h** | **24 h** | **P value*** |
| --- | --- | --- | --- | --- | --- | --- |
| OxPC | 10.0 ± 8.53 | 4.92 ± 3.13 | 6.55 ± 5.46 | 7.88 ± 6.54 | 8.40 ± 7.37 | 0.608 |
| OxPE | 0.188 ± 0.149 | 0.106 ± 0.066 | 0.197 ± 0.174 | 0.214 ± 0.219 | 0.281 ± 0.269 | 0.493 |
| OxPI | 1.14 ± 1.00 | 0.69 ± 0.43 | 1.08 ± 1.03 | 0.88 ± 0.66 | 1.17 ± 1.00 | 0.811 |
| OxPS | 0.024 ± 0.032 | 0.004 ± 0.002 | 0.007 ± 0.0068 | 0.013 ± 0.011 | 0.006 ± 0.005 | 0.147 |
| Total OxPL | 11.4 ± 9.64 | 5.72 ± 3.54 | 7.83 ± 5.94 | 8.99 ± 7.37 | 9.86 ± 8.63 | 0.636 |

Values are mean ± SD. Plasma from apo(a)-transgenic (n=3) and Lp(a)-transgenic (n=4) mice was pooled for LC-MS/MS analysis because this assay measures total plasma OxPL independently of carrier particle. *P values represent the overall effect of time by repeated-measures ANOVA. OxPG was not detected in mouse plasma.

**Supplementary Figure 1. Binding activity of murine IgG E06 to PC-BSA across two production batches.** Dose-response curves for E06 IgG from batches 5453 and 5205 were generated using serial dilutions of antibody and measuring relative light units (RLU) as a readout of antigen binding. Both batches demonstrated nearly overlapping sigmoidal curves with similar EC₅₀ values (0.360 nM and 0.404 nM), indicating consistent potency across production lots. Error bars represent mean ± SEM of replicate wells.

**Supplementary Figure 2.** **Relationship between lesional PC-OxPL area and macrophage area in aortic root atherosclerotic lesions.** Scatter plot showing the association between the percentage of plaque area positive for PC-OxPL and the percentage of plaque area occupied by macrophages in individual mice in the combined dataset. The original cohort included 30 mice; two placebo-treated mice died before study completion, leaving 28 mice for the principal analyses. Each symbol represents one mouse. The solid line represents the least-squares linear regression fit. Lesional PC-OxPL area correlated positively with macrophage area (Pearson *r* = 0.57, *P* = 0.004), indicating that plaques with greater PC-OxPL accumulation are associated with increased macrophage infiltration.

% Macrophage area

| % OxPL area | Percent_Macrophage_Area |
| --- | --- |
| 19.5721 | 38.9167 |
| 23.3898 | 17.3774 |
| 17.6381 | 53.1604 |
| 18.9703 | 45.105 |
| 21.5429 | 26.9303 |
| 15.5999 | 36.9069 |
| 24.3077 | 25.9245 |
| 19.5339 | 32.9417 |
| 24.924 | 31.3554 |
| 24.7924 | 31.1115 |
| 14.3441 | 49.7391 |
| 13.9978 | 23.34 |
| 25.8202 | 36.3599 |
| 13.3559 | 19.2698 |
| 8.96022 | 39.5026 |
| 20.5892 | 45.7402 |
| 22.7839 | 34.5319 |
| 19.2704 | 30.3566 |
| 29.4464 | 42.2697 |
| 11.7292 | 44.013 |
| 36.4585 | 62.819 |
| 43.6609 | 87.0278 |
| 28.2589 | 58.2888 |
| 33.1905 | 44.1966 |
| 27.0609 | 36.5607 |
| 18.0899 | 39.4933 |
